# Relationship between body mass index and physical fitness of children and adolescents in Xinjiang, China: a cross-sectional study

**DOI:** 10.1186/s12889-022-14089-6

**Published:** 2022-09-05

**Authors:** Guangwei Chen, Jianjun Chen, Jingzhi Liu, Yanyan Hu, Yang Liu

**Affiliations:** 1 Institute of Physical Education, Huaqiao University, Xiamen, 361021 China; 2 Qufu People’s Hospital, Qufu, 273100 China; 3 Department of Sports Teaching and Research, Xinjiang Institute of Engineering, Urumchi, 830023 China; 4 Institute of Physical Education, Xinjiang Normal University, Urumchi, 830054 China

**Keywords:** Obesity, Malnutrition, Health, U-shaped curve relationship, Weight status

## Abstract

**Background:**

Xinjiang is an economically underdeveloped area in China, but the obesity rate of children and adolescents is increasing year by year. Physical fitness and body mass index (BMI) are very important factors for healthy development, whereas few studies focus on the relationship between them in this region. This study aimed to explore the relationship between physical fitness and BMI of children and adolescents aged 7 to 18 in Xinjiang.

**Method:**

A total of 17,356 children and adolescents aged 7–18 years were involved. BMI was divided into five levels by percentiles, from very low to very high. Physical fitness was evaluated by five indicators: grip strength, standing long jump, sit-and-reach, 50 m dash, and endurance running. Single-factor analysis of variance was used to compare the Z-scores of the five physical fitness indicators among different BMI levels for the four age groups by gender. A nonlinear quadratic regression model was used to evaluate the relationship between BMI and each indicator in the four age groups.

**Result:**

There is a significant correlation between the five health-related indicators (grip strength, standing long jump, sit and reach, 50 m dash, endurance run) at two age groups (13-15 yrs., 16-18 yrs) of children and adolescents in Xinjiang, China. The range of the Pearson coefficient is 0.048 ~ 0.744. For the other two age groups (7-9 yrs., 10-12 yrs.,) significant correlations are found only in some indicators, and the Pearson coefficient ranges from 0.002 to 0.589. The relationship between BMI and physical fitness presents an U-shaped or inverted U-shaped curve in most age groups(R^2^ ranges from − 0.001 to 0.182. Children and adolescents with normal BMI score higher on physical fitness tests, and boys (R^2^ ranges from − 0.001 to 0.182) are more pronounced than girls (R^2^ ranges from 0.001 to 0.031).

**Conclusion:**

Children and adolescents with a BMI above or below the normal ranges have lower physical fitness than those with normal BMI. BMI and physical fitness have an U-shaped or inverted U-shaped curve relationship, and the impact is more evident in boys than girls. Targeted actions such as improving the quality of physical education classes, advocating students to keep a balanced diet and physical exercise should be taken designedly.

## Introduction

Meta-analysis suggested that anthropometric indicators such as body mass index (BMI), waist circumference, and waist-to-height ratio can be used by health professionals to assess body fat in children and adolescents [1]. BMI was widely used due to its simplicity, easy measurement, and high reliability [2, 3]. BMI is positively related to physical disorders such as hypertension, type 2 diabetes, and cardiovascular disease. It can also negatively affect the executive function, educational outcomes, and intellectual development of children and adolescents [4–6]. Conversely, wasting and malnutrition caused by low BMI are also harmful to the physical and mental health of children and adolescents [7, 8]. Therefore, the maintenance of a normal BMI is fundamental to the healthy development of children and adolescents.

As a major component of physical health, physical fitness is very important to the lives and learning of children and adolescents [9]. Physical fitness is a comprehensive indicator that closely reflects cardiopulmonary endurance, muscle strength, speed, and flexibility in children and adolescents [10]. Stodden et al. confirmed a positive correlation between physical fitness and the health of children and adolescents [11]. A study conducted by Barnett et al. also reported that good physical fitness was correlated with better health during childhood and adolescence, which could continue into adulthood and confer many health benefits in adults, indicating that physical fitness is of great significance for future healthy development [12].

The relationship between BMI and physical fitness has recently been extensively researched, but has mainly been viewed from three angles. Firstly, overweight/obese people have shown a negative linear relationship between BMI and fitness [2, 13]. Secondly, BMI is a potential covariate for fitness [14, 15]. Thirdly, relationship between the physical fitness and BMI during adolescence is quadratic [16, 17]. However, most of these studies focused on the effect of higher BMI on physical fitness, the effect of underweight or malnutrition due to lower BMI was seldom involved.

Much of the published research in this area has focused on children and adolescents in developed regions. However, with a higher prevalence of underweight, developing areas should be more concerned given their poor medical facilities [18]. Xinjiang Uygur Autonomous Region, located in the northwest of China, is one of the underdeveloped provinces of China [19]. In 2016, the rate of malnutrition among Kazakh children and adolescents in Xinjiang was 17%, higher than in developed areas of China (0.9%) [20]. We have clarified the benefit of normal BMI on the overall physical fitness among Xinjiang children and adolescents [21], but the effect of BMI on each physical fitness indicator remains unknown. Given the increased prevalence of obesity [22] and declined physical fitness levels [23] of Xinjiang children and adolescents since 1985, the present study hypothesized that there is a “U” or inverted “U”-shaped relationship between BMI and each physical fitness in Xinjiang children and adolescents.

## Materials and methods

### Data resources

Data were selected from the Chinese National Survey on Students’ Constitution and Health (CNSSCH), which is currently the largest national survey on the physical health of children and adolescents in China. This project is conducted every 5 years from 1985 to 2014 by the national administrative departments, including the Ministry of Education, Ministry of Science and Technology, National Civil Affairs Commission, Ministry of Finance, National Health Commission of the People’s Republic of China, and the General Administration of Sport of China. All student names were numerically coded to avoid leaking their personal information.

### Participants

Participants in the present study were selected from the CNSSCH project in 2014 involved children and adolescents age 7–18 years from the Xinjiang Uygur Autonomous Region, China. All the participants should have lived in Xinjiang for a minimum of 1 year and were required to undergo a simple examination before the test to ensure they are free from mental or physical illnesses. According to the arrangement of the State General Administration of Sport and the Ministry of Education of the People’s Republic of China, all the students in China have physical education classes 2–4 times a week and the students were organized to have one-hour collective physical exercise after class during weekdays without physical education classes.

This is a present situation research and the research variable belongs to counting data. Therefore, we used the sample estimation calculation formula as follows:

$$n=\frac{Za/{2}^2\times p\left(1-p\right)}{d^2}$$

$$d=0.15\times p,a=0.05\left(\mathrm{two}\ \mathrm{sides}\right),\kern0.5em Za/{2}^2=1.96$$

According to the Statistical Bulletin on educational Development of Xinjiang Uygur Autonomous Region in 2010, the population is 21,813,300 and there are 2,939,100 children and adolescents, resulting that p equals to 0.1347(2,939,100/21,813,300) and *n* = 1096. Provided 10% of missing data, the sample size should be 1206. The present study was conducted in six regions of Xinjiang. considering the urban and rural distribution, we tested 14,468 Xinjiang children and adolescents and obtained 17,356 valid data.

**Fig. 1:**
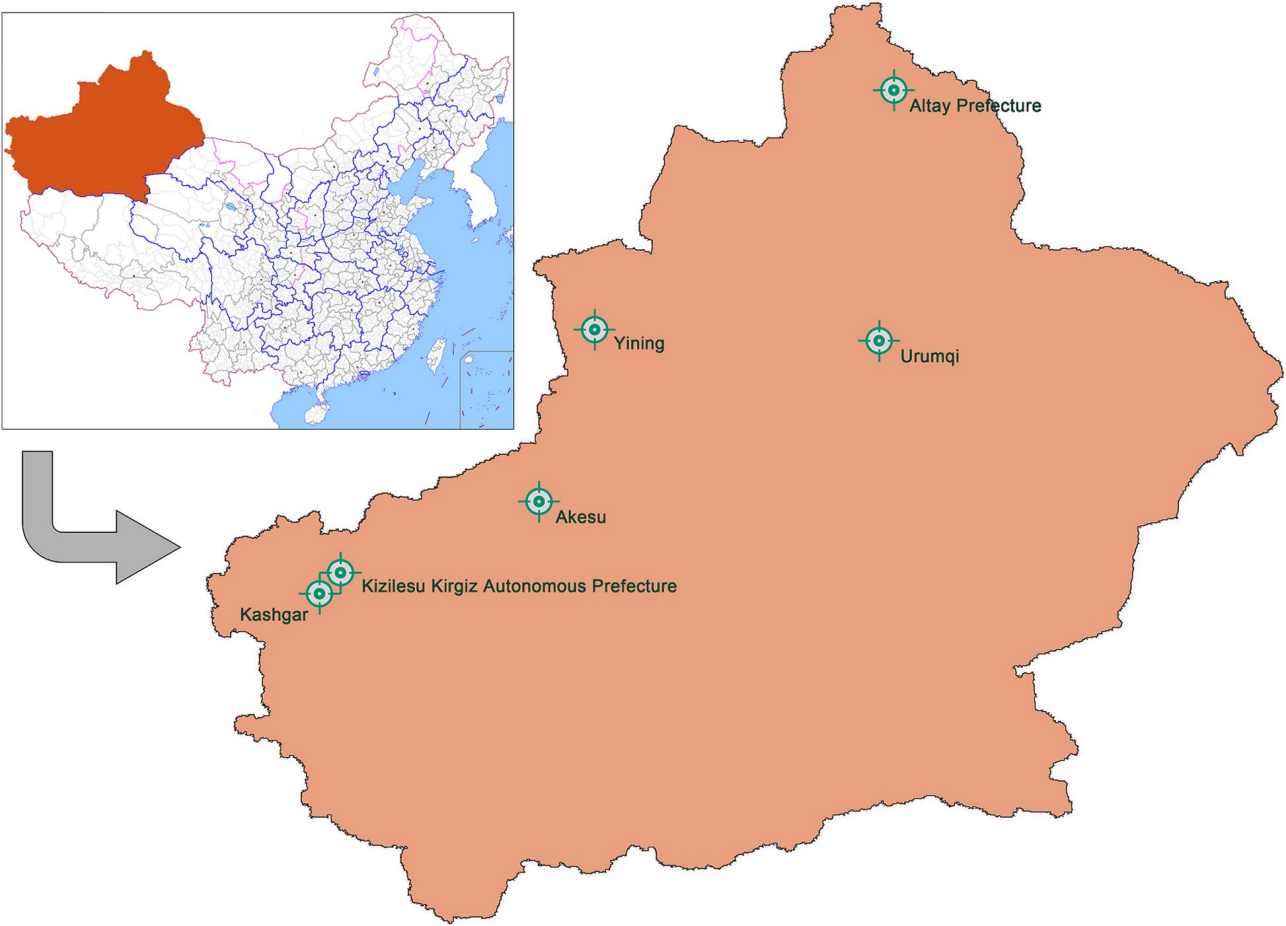
Sampling diagram for children and adolescents in Xinjiang, China

The recruitment procedure was divided into three stages: 1) Based on different levels of economic development and geographical distribution of Xinjiang Uygur Autonomous Region in China, six survey sites (Urumqi, Yining, Altay, Aksu, Kashi, and Atushi) were selected (Fig. 1) [24]; 2) Considering the large differences between urban and rural areas, 5 urban and 5 rural schools were selected as survey schools from each survey sites; 3) In each school, a stratified cluster sampling method was used to select classes from each grade, and students in the selected class were recruited as participants in the cluster. After excluding 768(4.24%) missing data, a total of 17,356 (boys 8671,49.96%) students were recruited as participants.

### Physical fitness test

Physical fitness indicators (height, weight, grip strength, standing long jump, sit-and-reach, 50 m dash, and endurance running) were tested by trained and qualified professional testers and each tester took charge of one test [21]. All test procedures were conducted according to CNSSCH guidelines, which have been proved to be validated for Chinese children and adolescents [21, 24]. To ensure the accuracy of test data and reduce errors reported by the different test times of the day, each test was carried out at a set time, either in the morning or in the afternoon. Height, weight, grip strength, standing long jump, sit-and-reach, 50 m dash were measured from ages 7 to 18 years. Endurance run included eight 50 m shuttle runs (for both boys and girls aged 7–12 years) and distance running (1000 m endurance running for boys aged 13–18 years, and 800 m endurance running for girls aged 13–18 years). BMI was calculated by weight (kg) / height (m^2^). The extreme values for each variable was defined as follows [24]: BMI ≤ 10 or > 40 kg/m^− 2^; grip strength < 1 kg or > 70 kg; standing long jump < 50 or > 300 cm; sit-and-reach ≤ − 8 or > 26 cm; 50 m dash < 6.0 or > 16.0 s; 800 m running < 140 or > 400 s; 1000 m running < 150 or > 370 s; 50 m × 8 round running < 60 or > 200 s.

To compare physical fitness of children and adolescents with different BMI levels, BMI was divided into five levels by percentile for both boys and girls: very low (BMI < 5 percentiles); low (5 ≤ BMI < 15 percentiles); normal (15 ≤ BMI < 85 percentiles); high (85 ≤ BMI < 95 percentiles); very high (BMI ≥ 95 percentiles) [21]. As a result, the numerical value of BMI for each group is as followed: for boys: very low (BMI < 14.67 kg/m^2^);low (14.67 kg/m^2^ ≤ BMI < 15.69 kg/m^2^); normal (15.69 kg/m^2^ ≤ BMI < 22.11 kg/m^2^); high (22.11 kg/m^2^ ≤ BMI < 25.02 kg/m^2^); very high (BMI ≥ 25.02 kg/m^2^); For girls: very low (BMI < 14.13 kg/m^2^); low (14.13 kg/m^2^ ≤ BMI < 15.27 kg/m^2^); normal (15.27 kg/m^2^ ≤ BMI < 22.09 kg/m^2^); high (22.09 kg/m^2^ ≤ BMI < 24.22 kg/m^2^); very high (BMI ≥ 24.22 kg/m^2^). Taking the mean and standard deviation (SD) of the corresponding gender and age as references, standardized Z-scores for BMI, grip strength, standing long jump, sit-and-reach, 50 m dash, and endurance running were calculated as Z- score = (measured value - reference value) / reference SD [24]. The participants were divided into four age groups according to age and gender: 7–9 years, 10–12 years, 13–15 years, and 16–18 years.

### Statistical analyses

We analyzed the Z-scores of fitness indicators for boys and girls at different levels of BMI across age and gender groups. Single-factor variance analysis and the least significant difference approach were used to compare the Z scores of each indicator between the different BMI levels by gender in the four age groups. Comparisons between groups are reflected by the effect size (Cohen’s d: small effect: 0.2; medium effect: 0.5; large effect: 0.8) [25]. A non-linear quadratic regression model was used to assess the association between BMI and fitness indicators in the age and gender groups. We performed regression analysis to establish the eq. Y = aX^2^ + bX + c (Y = Z-score of each physical fitness indicator, X = BMI Z-score), where a, b, and c are constants. Y was used as the dependent variable, and X was considered the independent variable. The level of statistical significance was set at 0.05, and all analyses were conducted using the statistical software SPSS version 23.0 (IBM, Armonk, NY, USA).

## Results

**Fig. 2:**
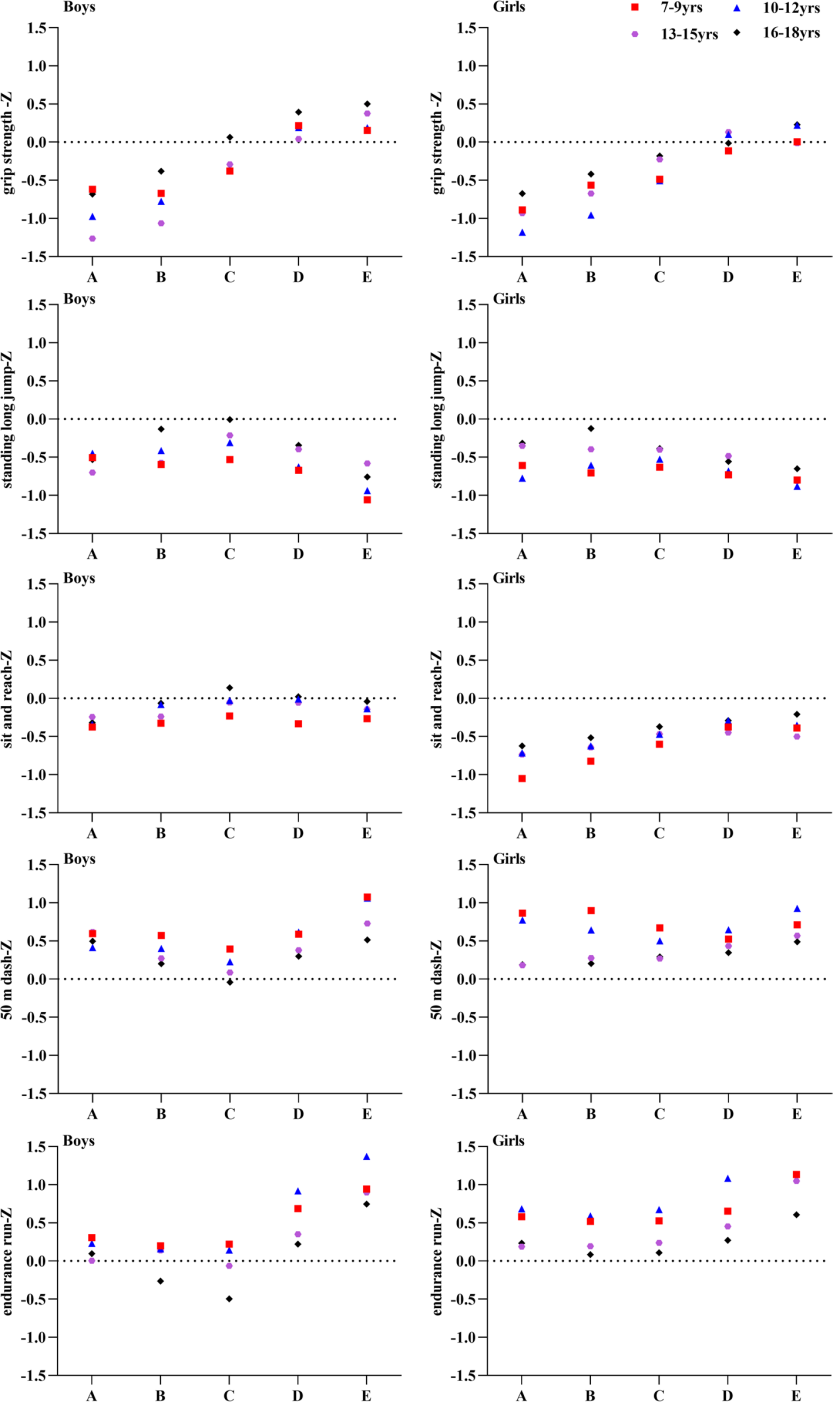
Z-scores for the five physical fitness indicators among children and adolescents with different BMI levels in Xinjiang, China. Note: BMI<5 Percentile(**A**); 5 ≤ BMI<15 Percentile(**B**); 15 ≤ BMI< 85 Percentile(**C**); 85 ≤ BMI < 95 Percentile(**D**); BMI ≥ 95 Percentile(**E**)

After excluding 752 participants (4.2%) because of missing data or extreme values, 17,356 children and adolescents (8671 boys and 8685 girls, Table 1) aged 7–18 years were recruited for the present study. Evaluation of BMI and physical fitness indicators of children and adolescents aged 7–18 in Xinjiang, China (Table 2). Overall, height, weight, BMI, grip strength, and standing jump increased with age, reaching their highest level in the 16–18 age group. The mean, SD, and Z-scores of the five fitness indicators with different BMI levels among boys (Table 3) and girls (Table 4) were compared, and the effect sizes between the different age groups were also calculated. Overall, children and adolescents with normal BMI performed best in standing long jump, sit-and-reach, 50 m dash, endurance running, and children and adolescents with low and very low BMI achieved better results than those with high BMI. The scores of grip strength gradually increased with BMI (Fig. 2).

**Table 1:** Sample distribution by gender and age for children and adolescents in Xinjiang, China

Age (yrs)	Boys	Girls	Total
**7**	739	740	1479
**8**	713	723	1436
**9**	740	723	1463
**10**	724	748	1472
**11**	725	646	1371
**12**	704	716	1420
**13**	686	731	1417
**14**	727	714	1441
**15**	727	721	1448
**16**	736	746	1482
**17**	726	742	1468
**18**	724	735	1459
**Total**	8671	8685	17,356

**Table 2:** The status of BMI and physical fitness of children and adolescents aged 7–18 in Xinjiang, China

Age (yrs)	n	Height (cm)	Weight (kg)	BMI (kg/m^**2**^)	Grip strength (kg)	Standing long jump (cm)	Sit and reach (cm)	50 m dash(s)	Endurance run(s)
**Boys**									
**7-9 yrs**	2192	128.05 ± 7.64	27.67 ± 6.14	16.71 ± 2.33	11.37 ± 3.36	121.31 ± 20.30	4.39 ± 4.63	11.04 ± 1.22	136.01 ± 16.14
**10-12 yrs**	2153	143.98 ± 8.93	38.31 ± 9.48	18.26 ± 3.04	17.54 ± 5.09	149.63 ± 20.59	4.14 ± 5.03	9.73 ± 1.00	122.85 ± 16.58
**13-15 yrs**	2140	162.55 ± 9.39	52.08 ± 10.91	19.55 ± 2.84	30.63 ± 8.69	191.27 ± 25.85	6.78 ± 6.05	8.34 ± 0.95	285.67 ± 45.09
**16-18 yrs**	2186	171.82 ± 6.34	62.45 ± 9.75	21.12 ± 2.85	41.87 ± 8.00	220.52 ± 24.51	11.11 ± 6.71	7.72 ± 0.82	253.79 ± 38.76
**Girls**									
**7-9 yrs**	2186	126.57 ± 7.99	25.89 ± 5.4	16.02 ± 2.01	9.56 ± 3.03	111.91 ± 19.73	6.69 ± 5.17	11.73 ± 1.26	143.40 ± 16.32
**10-12 yrs**	2110	144.58 ± 9.20	37.26 ± 8.84	17.61 ± 2.71	15.46 ± 4.90	134.91 ± 19.96	6.53 ± 5.41	10.45 ± 1.04	133.90 ± 25.34
**13-15 yrs**	2166	156.67 ± 6.18	48.99 ± 8.01	19.91 ± 2.72	22.15 ± 5.37	151.69 ± 20.39	8.21 ± 6.04	9.89 ± 1.11	272.26 ± 38.44
**16-18 yrs**	2223	159.13 ± 5.62	53.38 ± 6.92	21.07 ± 2.47	24.60 ± 6.35	158.64 ± 19.34	10.8 ± 5.91	10.01 ± 1.16	266.22 ± 37.27
**Total**									
**7-9 yrs**	4378	127.31 ± 7.85	26.78 ± 5.85	16.37 ± 2.20	10.47 ± 3.32	116.62 ± 20.56	5.54 ± 5.04	11.38 ± 1.29	139.70 ± 16.64
**10-12 yrs**	4263	144.28 ± 9.07	37.79 ± 9.18	17.94 ± 2.90	16.51 ± 5.10	142.35 ± 21.57	5.32 ± 5.35	10.09 ± 1.08	128.32 ± 22.07
**13-15 yrs**	4306	159.59 ± 8.46	50.53 ± 9.68	19.73 ± 2.79	26.37 ± 8.37	171.36 ± 30.54	7.50 ± 6.09	9.12 ± 1.29	278.92 ± 42.40
**16-18 yrs**	4409	165.42 ± 8.72	57.87 ± 9.58	21.10 ± 2.67	33.16 ± 11.25	189.32 ± 38.00	10.96 ± 6.32	8.87 ± 1.52	260.06 ± 38.51

*M* Mean, *SD* Standard Deviation, *BMI* Body mass index

**Table 3:** Comparison of Z-scores for the five physical fitness indicators among different BMI levels for boys in Xinjiang, China

Tests	Age (yrs)	BMI<5th(A)		5th ≤ BMI<15th(B)		15th ≤ BMI<85th (C)		85th ≤ BMI<95th (D)		BMI ≥ 95th (E)		Cohen’s ***d*** ^***#***^									
		N	Mean (SD)	N	Mean (SD)	N	Mean (SD)	N	Mean (SD)	N	Mean (SD)	A/B	A/C	A/D	A/E	B/C	B/D	B/E	C/D	C/E	D/E
**grip strength**	**7-9 yrs**	108	−0.619 (1.018)	220	− 0.671 (0.966)	1537	− 0.38 (0.998)	218	0.215 (1.045)	109	0.153 (0.985)	0.1	0.2^a^	0.8^a^	0.8^a^	0.3^a^	0.9^a^	0.8^a^	0.6^a^	0.5^a^	0.1
	**10-12 yrs**	107	−0.974 (0.738)	213	−0.776 (0.780)	1512	−0.369 (0.965)	213	0.191 (0.987)	108	0.189 (0.940)	0.3	0.7^a^	1.3^a^	1.4^a^	0.5^a^	1.1^a^	1.1^a^	0.6^a^	0.6^a^	0.0
	**13-15 yrs**	106	−1.265 (0.790)	214	−1.064 (0.802)	1499	−0.292 (1.006)	215	0.041 (1.116)	106	0.376 (1.078)	0.3	0.9^a^	1.4^a^	1.7^a^	0.8^a^	1.1^a^	1.5^a^	0.3^a^	0.6^a^	0.3^a^
	**16-18 yrs**	108	−0.682 (0.933)	219	−0.382 (0.942)	1532	0.063 (0.974)	219	0.393 (1.039)	108	0.502 (0.949)	0.3^a^	0.8^a^	1.1^a^	1.3^a^	0.5^a^	0.8^a^	0.9^a^	0.3^a^	0.5^a^	0.1
**standing long jump**	**7-9 yrs**	108	−0.505 (0.942)	220	− 0.597 (0.912)	1537	− 0.532 (0.974)	218	− 0.670 (0.974)	109	−1.059 (0.981)	0.1	0.0	0.2	0.6^a^	0.1	0.1	0.5^a^	0.1^a^	0.5^a^	0.4^a^
	**10-12 yrs**	107	−0.448 (0.789)	213	−0.414 (0.836)	1512	−0.310 (0.884)	213	−0.627 (0.945)	108	−0.939 (0.826)	0.0	0.2	0.2	0.6^a^	0.1	0.2^a^	0.6^a^	0.3^a^	0.7^a^	0.4^a^
	**13-15 yrs**	106	−0.700 (0.761)	214	−0.574 (0.847)	1499	−0.215 (0.915)	215	−0.397 (1.005)	106	−0.580 (0.930)	0.2	0.6^a^	0.3^a^	0.1	0.4^a^	0.2^a^	0.0	0.2^a^	0.4^a^	0.2
	**16-18 yrs**	108	−0.531 (1.030)	219	−0.131 (0.986)	1532	−0.007 (1.008)	219	−0.345 (0.918)	108	−0.758 (0.976)	0.4^a^	0.5^a^	0.2	0.2	0.1	0.2^a^	0.6^a^	0.4^a^	0.8^a^	0.4^a^
**sit and reach**	**7-9 yrs**	108	−0.375 (0.790)	220	−0.325 (0.760)	1537	−0.231 (0.815)	218	−0.333 (0.878)	109	−0.267 (0.880)	0.1	0.2	0.1	0.1	0.1	0.0	0.1	0.1	0.0	0.1
	**10-12 yrs**	107	−0.341 (0.755)	213	−0.082 (0.737)	1512	−0.028 (0.799)	213	−0.010 (0.834)	108	−0.135 (0.802)	0.3^a^	0.4^a^	0.4^a^	0.3	0.1	0.1	0.1	0.0	0.1	0.2
	**13-15 yrs**	106	−0.243 (0.721)	214	−0.238 (0.796)	1499	−0.054 (0.837)	215	−0.056 (0.91)	106	−0.144 (0.853)	0.0	0.2^a^	0.2	0.1	0.2^a^	0.2^a^	0.1	0.0	0.1	0.1
	**16-18 yrs**	108	−0.318 (0.920)	219	−0.064 (0.865)	1532	0.138 (0.908)	219	0.022 (0.913)	108	−0.042 (0.939)	0.3^a^	0.5^a^	0.4^a^	0.3^a^	0.2^a^	0.1	0.0	0.1	0.2^a^	0.1
**50 m dash**	**7-9 yrs**	108	0.596 (1.032)	220	0.572 (1.120)	1537	0.394 (1.020)	218	0.589 (1.063)	109	1.077 (1.193)	0.0	0.2	0.0	0.4^a^	0.2^a^	0.0	0.4^a^	0.2^a^	0.6^a^	0.4^a^
	**10-12 yrs**	107	0.413 (0.927)	213	0.400 (1.064)	1512	0.227 (0.968)	213	0.617 (0.914)	108	1.060 (1.074)	0.0	0.2	0.2	0.6^a^	0.2^a^	0.2^a^	0.6^a^	0.4^a^	0.8^a^	0.4^a^
	**13-15 yrs**	106	0.615 (1.061)	214	0.271 (0.874)	1499	0.086 (0.993)	215	0.380 (1.119)	106	0.730 (1.326)	0.4^a^	0.5^a^	0.2	0.1	0.2^a^	0.1	0.4^a^	0.3^a^	0.5^a^	0.3^a^
	**16-18 yrs**	108	0.496 (1.137)	219	0.201 (1.029)	1532	−0.041 (0.956)	219	0.298 (1.072)	108	0.515 (1.138)	0.3^a^	0.5^a^	0.2	0.0	0.2^a^	0.1	0.3^a^	0.3^a^	0.5^a^	0.2
**endurance run**	**7-9 yrs**	108	0.307 (0.963)	220	0.198 (0.911)	1537	0.220 (0.906)	218	0.685 (1.040)	109	0.944 (1.047)	0.1	0.1	0.4^a^	0.6^a^	0.0	0.5^a^	0.8^a^	0.5^a^	0.7^a^	0.2^a^
	**10-12 yrs**	107	0.231 (0.926)	213	0.158 (0.917)	1512	0.144 (0.898)	213	0.918 (1.017)	108	1.372 (0.927)	0.1	0.1	0.7^a^	1.2^a^	0.0	0.8^a^	1.3^a^	0.8^a^	1.3^a^	0.5^a^
	**13-15 yrs**	106	0.004 (0.934)	214	0.140 (1.001)	1499	−0.064 (0.992)	215	0.351 (1.081)	106	0.898 (1.014)	0.1	0.1	0.3^a^	0.9^a^	0.2^a^	0.2^a^	0.8^a^	0.4^a^	1.0^a^	0.5^a^
	**16-18 yrs**	108	0.097 (1.140)	219	−0.263 (1.125)	1532	− 0.495 (1.027)	219	0.220 (1.043)	108	0.745 (1.258)	0.3^a^	0.5^a^	0.1	0.5^a^	0.2^a^	0.4^a^	0.8^a^	0.7^a^	1.1^a^	0.5^a^

*M* Mean, *SD* Standard Deviation, *BMI* Body mass index

^#^ effect size between different groups; ^a^ *P* < 0.05. A/grip strength; B/standing long jump; C/sit and reach; D/50 m dash; E/endurance run

**Table 4:** Comparison of Z-scores for the five physical fitness indicators among different BMI levels for girls in Xinjiang, China

Tests	Age (yrs)	BMI<5th (A)		5th ≤ BMI<15th (B)		15th ≤ BMI<85th (C)		85th ≤ BMI<95th (D)		BMI ≥ 95th (E)		Cohen’s ***d*** ^***#***^									
		N	Mean (SD)	N	Mean (SD)	N	Mean (SD)	N	Mean (SD)	N	Mean (SD)	A/B	A/C	A/D	A/E	B/C	B/D	B/E	C/D	C/E	D/E
**grip strength**	**7-9 yrs**	109	−0.889 (0.886)	218	− 0.564 (0.882)	1532	−0.486 (1.017)	218	−0.115 (1.051)	109	0.005 (1.066)	0.4^a^	0.4^a^	0.8^a^	0.9^a^	0.1	0.5^a^	0.6^a^	0.4^a^	0.5^a^	0.1
	**10-12 yrs**	104	−1.182 (0.950)	212	−0.956 (0.833)	1478	−0.506 (1.050)	212	0.102 (1.116)	104	0.221 (1.209)	0.3	0.7^a^	1.2^a^	1.3^a^	0.5^a^	1.1^a^	1.1^a^	0.6^a^	0.6^a^	0.1
	**13-15 yrs**	108	−0.932 (0.913)	215	−0.674 (0.915)	1520	−0.226 (1.105)	216	0.132 (1.247)	107	−0.013 (1.181)	0.3^a^	0.7^a^	1.0^a^	0.9^a^	0.4^a^	0.7^a^	0.6^a^	0.3^a^	0.2	0.1
	**16-18 yrs**	109	−0.674 (1.064)	225	−0.419 (1.304)	1556	−0.182 (1.231)	223	−0.013 (1.496)	110	0.229 (1.248)	0.2	0.4^a^	0.5^a^	0.8^a^	0.2^a^	0.3^a^	0.5^a^	0.1	0.3^a^	0.2
**standing long jump**	**7-9 yrs**	109	−0.609 (1.019)	218	−0.706 (1.043)	1532	−0.631 (0.998)	218	−0.730 (1.025)	109	−0.798 (0.862)	0.1	0.0	0.1	0.2	0.1	0.0	0.1	0.1	0.2	0.1
	**10-12 yrs**	104	−0.776 (0.980)	212	−0.605 (0.916)	1478	−0.527 (0.971)	212	−0.680 (1.019)	104	−0.881 (1.053)	0.2	0.3^a^	0.1	0.1	0.1	0.1	0.3^a^	0.2^a^	0.3^a^	0.2
	**13-15 yrs**	108	−0.352 (1.013)	215	−0.396 (1.003)	1520	−0.402 (1.000)	216	−0.484 (0.975)	107	−0.812 (1.074)	0.0	0.0	0.1	0.4^a^	0.0	0.1	0.4^a^	0.1	0.4^a^	0.3^a^
	**16-18 yrs**	109	−0.317 (0.983)	225	−0.125 (0.943)	1556	−0.387 (0.983)	223	−0.557 (1.011)	110	−0.650 (0.937)	0.2	0.1	0.2^a^	0.3^a^	0.3^a^	0.4^a^	0.6^a^	0.2^a^	0.3^a^	0.1
**sit and reach**	**7-9 yrs**	109	−1.051 (0.822)	218	−0.824 (0.899)	1532	−0.600 (0.927)	218	−0.375 (0.969)	109	−0.388 (0.988)	0.3^a^	0.5^a^	0.8^a^	0.7^a^	0.2^a^	0.5^a^	0.5^a^	0.2^a^	0.2^a^	0.0
	**10-12 yrs**	104	−0.717 (0.833)	212	−0.624 (0.800)	1478	−0.471 (0.873)	212	−0.292 (0.808)	104	−0.350 (0.871)	0.1	0.3^a^	0.5^a^	0.4^a^	0.2^a^	0.4^a^	0.3^a^	0.2^a^	0.1	0.1
	**13-15 yrs**	108	−0.736 (0.719)	215	−0.644 (0.825)	1520	−0.468 (0.882)	216	−0.450 (0.925)	107	−0.501 (0.868)	0.1	0.3^a^	0.3^a^	0.3^a^	0.2^a^	0.2^a^	0.2	0.0	0.0	0.1
	**16-18 yrs**	109	−0.623 (0.804)	225	−0.515 (0.941)	1556	−0.370 (0.871)	223	−0.292 (0.858)	110	−0.209 (0.867)	0.1	0.3^a^	0.4^a^	0.5^a^	0.2^a^	0.2^a^	0.3^a^	0.1	0.2	0.1
**50 m dash**	**7-9 yrs**	109	0.865 (1.158)	218	0.898 (1.190)	1532	0.671 (1.079)	218	0.524 (0.871)	109	0.712 (1.023)	0.0	0.2	0.3^a^	0.1	0.2^a^	0.4^a^	0.2	0.1	0.0	0.2
	**10-12 yrs**	104	0.774 (1.208)	212	0.644 (0.998)	1478	0.501 (1.049)	212	0.645 (1.039)	104	0.925 (1.109)	0.1	0.2^a^	0.1	0.1	0.1	0.0	0.3^a^	0.1	0.4^a^	0.3^a^
	**13-15 yrs**	108	0.182 (1.001)	215	0.276 (1.104)	1520	0.268 (1.089)	216	0.434 (1.126)	107	0.570 (1.163)	0.1	0.1	0.2	0.4^a^	0.0	0.1	0.3^a^	0.1	0.3^a^	0.1
	**16-18 yrs**	109	0.189 (1.000)	225	0.205 (1.072)	1556	0.291 (1.046)	223	0.345 (1.061)	110	0.488 (1.044)	0.0	0.1	0.2	0.3^a^	0.1	0.1	0.3^a^	0.1	0.2	0.1
**endurance run**	**7-9 yrs**	109	0.582 (1.079)	218	0.518 (1.021)	1532	0.527 (1.018)	218	0.653 (0.986)	109	1.133 (1.534)	0.1	0.1	0.1	0.4^a^	0.0	0.1	0.5^a^	0.1	0.5^a^	0.4^a^
	**10-12 yrs**	104	0.685 (1.221)	212	0.592 (1.311)	1478	0.674 (1.571)	212	1.084 (1.695)	104	1.618 (2.745)	0.1	0.0	0.3^a^	0.4^a^	0.1	0.3^a^	0.5^a^	0.3^a^	0.4^a^	0.2^a^
	**13-15 yrs**	108	0.188 (0.841)	215	0.195 (1.074)	1520	0.239 (1.070)	216	0.454 (1.001)	107	1.049 (1.092)	0.0	0.1	0.3^a^	0.9^a^	0.0	0.2^a^	0.8^a^	0.2^a^	0.7^a^	0.6^a^
	**16-18 yrs**	109	0.234 (1.180)	225	0.084 (1.239)	1556	0.109 (1.136)	223	0.271 (1.098)	110	0.605 (1.222)	0.1	0.1	0.0	0.3^a^	0.0	0.2	0.4^a^	0.1^a^	0.4^a^	0.3^a^

*M* Mean, *SD* Standard Deviation, *BMI* Body mass index

^#^ effect size between different groups; ^a^ *P* < 0.05. A/grip strength; B/standing long jump; C/sit and reach; D/50 m dash; E/endurance run

**Fig. 3:**
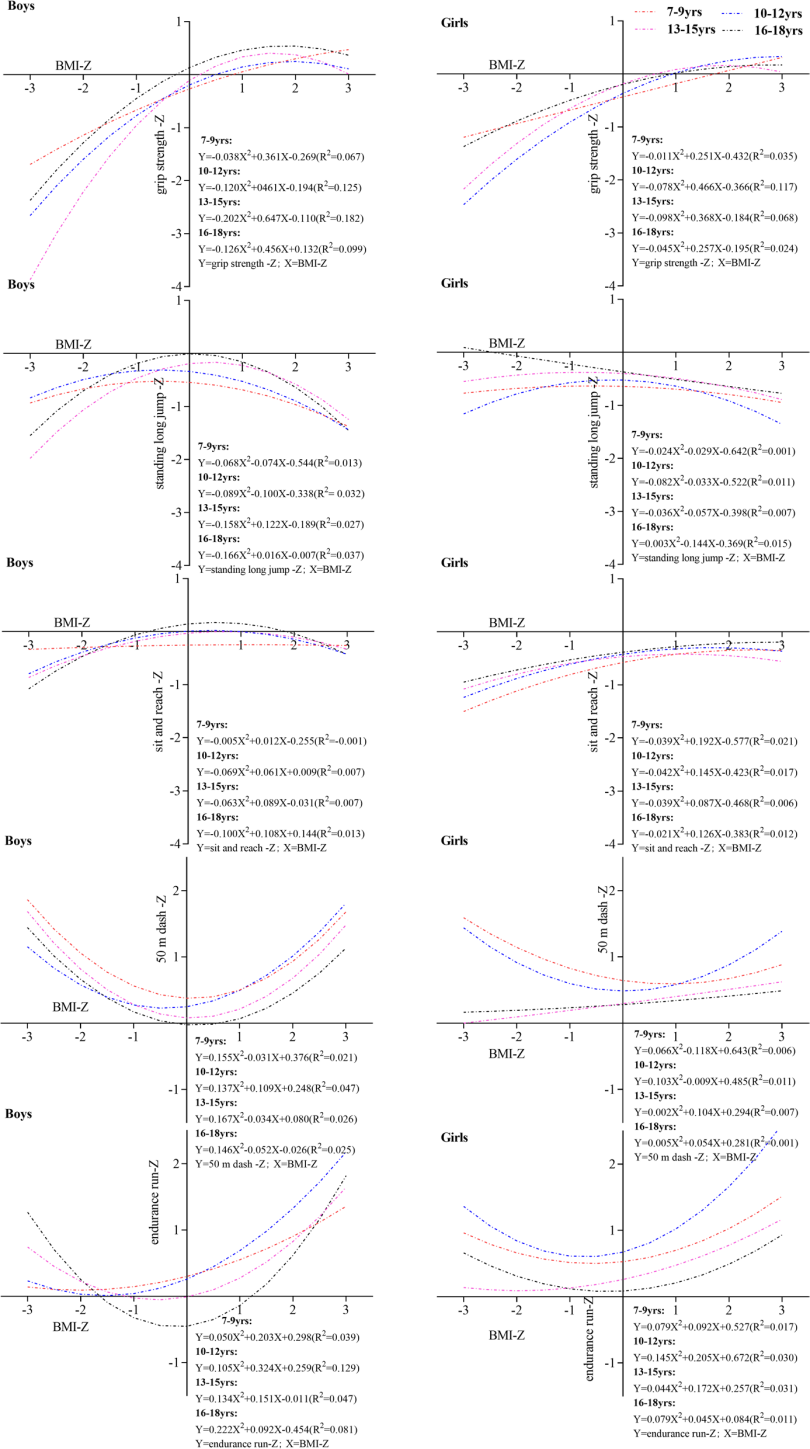
Association between BMI Z-scores and the five physical fitness items in the four age groups

The association between BMI Z-scores and Z-scores of the five fitness tests in the four age groups for boys and girls was presented in Fig. 3. Overall, for most age groups, the association presented as an inverted U - curve in grip strength (R^2^ ranges from 0.024 to 0.182), standing long jump (R^2^ ranges from 0.001 to 0.037), and sit-and-reach (R^2^ ranges from − 0.001 to 0.021). Whereas for the 50 m dash (R^2^ ranges from 0.001 to 0.047) and endurance running (R^2^ ranges from 0.001 to 0.129), it presented as a U - curve since lower values mean better performance. The inverted U-curve and the U-curve indicated that performance was best for children and adolescents with normal BMI. Whereas children and adolescents with a BMI higher or lower than the normal range resulted in lower performance. Children and adolescents with normal BMI score higher on physical fitness tests, and boys (R^2^ ranges from − 0.001 to 0.182) are more pronounced than girls (R^2^ ranges from 0.001 to 0.031).

## Discussion

The presented study estimated the relationship between BMI and physical fitness for children and adolescents in Xinjiang, China. We found that the physical fitness performance (grip strength, standing long jump, sit-and-reach, 50 m dash, and endurance running) in Xinjiang children and adolescents aged 7–18 years increased with age. Our results are consistent with findings among children and adolescents in China [26], Brazil [27], and adults in Germany [28]. The association between the BMI Z-score and Z-score of grip strength, standing long jump, and sit-and-reach showed an inverted U-curve, which was consistent with the results from Casonatto [29] and Gulías [30]. The association between BMI Z- score and Z-score of 50 m dash and endurance running showed a U-curve, which was in line with the conclusions of Li [31] and Huang [32]. Children and adolescents with normal BMI had the best performance in physical fitness. Whereas children and adolescents with a BMI above or below the normal range performed poorly. With regard to gender differences, the impact of BMI on fitness was more evident among boys than girls, consistent with the findings of Dong et al. [26] and Santos et al. [33].

Grip strength and standing long jump reflect the muscle strength of the upper and lower limbs, respectively. The association between BMI and grip strength of children and adolescents in the presented study was curvilinear, but almost linear in girls and young boys. The curvilinear association within boys aged 13–15 and 16–18 was more obvious, while in the 7–9 age group, the association was almost linear. These findings suggest that BMI has a stronger influence on grip strength among older Xinjiang children and adolescents. However, our results were inconsistent with the results of the study by Zaqout et al. [34], which suggested that the association between BMI and grip strength was linear, and grip strength performance was better in children and adolescents with higher BMI. One possible reason is that grip strength does not require support or movement of body weight. Boys with high BMI can be much stronger and more powerful, leading to better grip strength compared to their peers [35].

Our results also showed that BMI has a curvilinear association with standing jump, which is consistent with the studies on European children and adolescents [34]. We also found that BMI had a more obvious impact on standing long jump in boys than in girls, which is consistent with the research by Kwiecinski et al. [16] in Polish youth. Whereas the association between BMI and standing jump performance in girls aged 16–18 in our study tended to be linear. The different associations between boys and girls can be explained by the difference in muscular content in different genders.

Compared with other physical fitness, the performance of sit-and-reach was less affected by BMI, especially among girls. Unlike other physical fitness, sit-and-reach is not significantly affected by overweight since such activities do not have to overcome the resistance bought by high weight, and thus equally attractive to both underweight and overweight children and adolescents [36].

The 50 m dash and endurance running reflect speed ability and cardiorespiratory endurance level, respectively. Our results showed that the influence of BMI on 50 m dash performance in children and adolescents aged 7–18 in Xinjiang was more evident in boys than in girls. Girls aged 13–15 and 16–18 years showed a nearly positive linear relationship. Whereas girls aged 7–9 and 10–12 and boys aged 7–18 had a clear association in the U-curve. These results are consistent with the results for Lopes [37] and Rodrigues [38]. A possible explanation for this can be the fact that lower BMI means less muscle mass in adolescents, which can affect speed. Participants with higher BMI should overcome their resistance to weight, which results in a lower performance of the 50 m dash. BMI and endurance running performance in our study showed a clear association of the U - curve for boys and girls aged 7–18 years, which was consistent with previous studies [16, 17, 39–41]. However, the results should be interpreted with caution, since overweight or obese persons with a high BMI must overcome greater resistance to weight during the test [39, 42]. Artero et al. [35] found that, after adjusting fat mass, the association between 10 m × 4 round running test performance and weight status in girls became non-significant, which was signed before adjustment.

The results of this study found out an inverted U-shaped or U-shaped curve relationship between BMI and physical fitness in Xinjiang children and adolescents. Given the importance of physical fitness, children and adolescents can keep fit by maintaining a reasonable and normal BMI, thus reducing the incidence of disease caused by low physical fitness. Therefore, to improve physical fitness, targeted actions should be developed to address BMI-related effects in children and adolescents in Xinjiang. For example, the Physical Education and Health Curriculum Model of China, which was widely recognized in the field of physical education in China [43–45], should be carried out to help students maintain a normal BMI; Health courses also should be included in schools to make children and adolescents aware that they should keep a balanced diet and regular exercise. The government, communities, and families should also be united to take health promotion measures to keep the BMI of children and adolescents within the normal range [46].

There are some strengths in this study. The first strength is the large provincial representative sample, which has improved the objectivity and accuracy of the results, which has provided help to promote the healthy development of children and adolescents in Xinjiang, China. The second strength is that our study gives a picture of the patterns of how physical fitness changes with BMI in Xinjiang children and adolescents throughout the age (from 7 to 18 years old). However, there are also many limitations. The first limitation lies in the cross-sectional design which prevented the drawing of causal conclusions. Addressing the increasing prevalence of obesity and reduced fitness among children and adolescents, longitudinal studies are still needed to make causal inferences possible. The second limit is that, except for age and gender, we did not take into account other determinants of physical fitness (e.g. physical activity). The third limitation of the study is that we only measured five commonly used physical fitness and more accurate measures such as body composition were not included. The fourth limitation is that we didn’t control maturity status. This research also has some practical application value. First, it provides basis for the physical health intervention for children and adolescents in Xinjiang Uygur Autonomous Region, China. Secondly, it provides a reference for the government to formulate local public health policies and education policies in the future.

## Conclusions

This study evaluated the relationship between BMI and fitness in a large sample of children and adolescents in Xinjiang, China. Our result suggested BMI and physical fitness have an inverted U-shaped or U-shaped curve relationship in children and adolescents in Xinjiang, China, that is to say, children and adolescents with a BMI above or below the normal ranges have lower physical fitness than those with normal BMI. Therefore, to improve physical fitness, targeted actions should be developed to address BMI-related effects in children and adolescents in Xinjiang.

## Data Availability

All data generated or analysed during this study are included in supplementary information files.

## Abbreviations

BMI: Body Mass Index
CNSSCH: Chinese National Survey on Students’ Constitution and Health
SD: Standard Deviation
